# Trauma, Aggression, and Post Conflict Perpetration of Community Violence in Female Former Child Soldiers—A Study in Eastern DR Congo

**DOI:** 10.3389/fpsyt.2020.533357

**Published:** 2020-10-02

**Authors:** Katy Robjant, Sabine Schmitt, Amani Chibashimba, Samuel Carleial, Thomas Elbert, Anke Koebach

**Affiliations:** ^1^ Department of Psychology, University of Konstanz, Konstanz, Germany; ^2^ vivo international e.V., Konstanz, Germany

**Keywords:** appetitive aggression, conflict, female child soldiers, posttraumatic stress disorder, trauma, violence, DR Congo

## Abstract

**Objective:**

Former combatants are exposed to multiple traumatic stressors during conflict situations and usually participate in perpetration of violence. Ongoing perpetration of violence in post conflict areas, linked to mental health problems and appetitive aggression, destabilises peace keeping efforts. The aim of this study is to investigate lifetime exposure to violence and the relationship between this exposure and mental health and current violent behaviour in a sample of female former child soldiers with a history of perpetration of violence in Eastern DR Congo.

**Methods:**

98 female former child soldiers who had been abducted and forcibly recruited during the M23 insurgency (2012–2014) were assessed for lifetime exposure to trauma including perpetration of violence, clinical outcomes (PTSD and appetitive aggression), and current violent behaviour.

**Results:**

Female former child soldiers had been exposed to extremely high levels of trauma including perpetration of violence and presented with high levels of mental health problems. Linear regression models showed that current violent behaviour was predicted by both PTSD and appetitive aggression.

**Conclusions:**

Trauma exposure predicts ongoing perpetration of violence post conflict *via* the resulting mental health problems. The findings imply that if PTSD and appetitive aggression symptoms are successfully treated, ongoing violent behaviour in the community post conflict will also decrease.

## Introduction

Members of armed groups involved in combat, have experienced multiple adverse and often life-threatening events and also participated in the perpetration of violent acts. The more traumatic stressors the combatant experiences, the greater the risk of developing psychological problems (1) and the lower the likelihood of spontaneous remission (2), regardless of whether this exposure occurred within military or para-military contexts, low- or high-income settings. Mental health problems including posttraumatic stress disorder (PTSD), depression, suicidality, and substance use disorders (SUD) are prevalent among combatants who return to high-income settings having served in the military (3–8), as well as in combatants in military and paramilitary groups in low- and middle-income conflict and post conflict settings (9–15).

Across settings, leaving the battle ground and retuning to civilian life is not straightforward. Post conflict adjustment difficulties, including ongoing aggressivity in some former combatants, have been documented since the US/Vietnam war [e.g., (16)]. Research has also shown that the perpetration of violence may continue in civilian post conflict settings (17–20). At the societal level, within unstable contexts, previous experience of combat interferes with peacekeeping efforts since combatants continue to perpetrate violence, causing further destabilisation (21–23).

Violence perpetrated by former combatants in civilian post conflict settings is related in part to mental health problems that result from conflict experiences. The presence of PTSD symptoms, and in particular hyperarousal symptoms and risk perception, may lead to reactive aggression (24, 25), as may substance misuse (26), as well as a combination of alcohol misuse and PTSD (5, 27, 28).

Within settings in which neither societal, political nor military sanctions impose restrictions on the types of violence perpetrated, extreme violence has been reported within military, paramilitary and criminal organisations [e.g., (29–32)]. Severe violence against civilians has been described as characteristic of “new wars” (33). Todays armed conflicts often have a ‘hybrid’ quality to them (34), whereby the distinction between periods of war and peace is blurred. Regions are trapped in an ongoing cycle of transition attempts with outbreaks of sporadic fighting and high levels of criminality. Former combatants acting within and outside of their para military networks contribute to the maintenance of instability during this period (35). Additional psychological frameworks are required for understanding the perpetration of this severe violence, particularly post conflict when violence should no longer be desired or required. Appetitive aggression, i.e., the intrinsic enjoyment of violence, which has been called into play by the very fact of having perpetrated atrocities, has been proposed as an explanation for the continued perpetration of extreme violence in such circumstances [e.g., (36)].

Individuals with high appetitive aggression are drawn towards recalling, planning, witnessing and continuing to perpetrate acts of aggression for personal gratification and satisfaction (37, 38), distinct neural circuitry underlies appetitive and reactive aggression (39, 40). Hemmings and colleagues showed in gang members that different serotonin receptor genes were involved in the two types of aggression (although the two are frequently activated in parallel) (41). Therefore, appetitive aggression differs at a biological level to reactive aggression and is hypothesised to be related to histories of violence perpetration.

Given that such extreme violence is often found within unstable and traumatic settings, the relationship between posttraumatic stress symptoms and appetitive aggression is of importance. This relationship is theorised to result from the integration of perpetrated violence into the associative trauma networks which underlie the memory disorder characterising PTSD (42, 43). Amongst trauma exposed individuals, acts of perpetrated violence will share common cues with other traumatic events, although they will be associated with positive thoughts and feelings rather than negative ones. Perpetrated events are connected within an associative memory, called a “hunting network” (38) and when recalled, will result in positive feelings towards violence. Since many of the cues are similar within the hunting network and the trauma network, i.e., the associative memory of traumatic experiences, engaging in violence may cause the hunting network to be triggered, rather than the trauma network. This means that positive rather than negative feelings will be experienced, and violence will be more likely to be sought either within the imagination or through actual perpetration (36, 38). Previous work has shown a protective effect of appetitive aggression for PTSD (44), suggesting an implicit motivation for the continuing perpetration of violence as means to avoid the distressing symptoms of PTSD. However, this relationship only holds to a certain threshold of trauma exposure (45, 46).

Among male non-combatants, the level of appetitive aggression is low, and among female non-combatants it is hardly known. However, once adults become fighters, the same level of substantial appetitive aggression can be seen in both males and females (47). While the relationship between violence exposure and mental health per se may be similar in male and female samples, recent investigations have suggested distinct trajectories to aggression for female (ex)combatants, e.g., in interaction with childhood adversities (20). Separate studies for male and female survivors of combat are therefore required to understand the needs of these individuals and successfully mitigate the consequences of violence at the individual and societal levels.

With the ongoing conflict in Eastern DRC, extreme levels of trauma are experienced and perpetrated. Sexual violence is highly prevalent, and it is associated with poor mental and physical health outcomes (11, 48–50). Combatants, including females, can both perpetrate and be victim of rape and sexual violence (51, 52). For example, female former child soldiers explained to our research group that they frequently trapped other women and girls in situations where they could be raped by men, or even participated in the physical restraint of other women and girls, the motivation apparently being to reduce the number of women and girls who had not been raped in order to diffuse the stigma and social exclusion that they themselves were experiencing as known victims of rape. Social stigma and marginalisation is commonly encountered by returning female child soldiers (53), and the lack of social acknowledgement and community exclusion may further impact on psychopathology and recovery (54).

Sensitive periods within development may result in a more problematic impact of trauma exposure and perpetration of violence in youth in terms of the likelihood of development of psychopathology (55) and appetitive aggression (56). Studies of psychological problems within former child soldiers support this (13, 57, 58).

While some studies have shown the relationship between exposure to violence and mental health outcomes including appetitive aggression, evidence has so far been restricted to male samples. In this study, we aimed to investigate exposure to violence and the relationship between this exposure and mental health outcomes in a sample of female former child soldiers with a history of abduction and perpetrated violence within the M23 war (2012–2014). Those with a PTSD diagnosis were allocated for treatment in a trial reported elsewhere (59). As we did not want to deprive adolescents of treatment, we also offered women aged 16–18 years the opportunity to participate in these investigations.

## Material and Methods

### Procedure

Participants were recruited through PAMI, an NGO in Kibumba, Eastern DR Congo. Participants of this study were part of a larger sample recruited into an RCT described in (59). PAMI offer practical support, mediation and counselling for young people affected by conflict and their families. All women who were at least 16 years of age and had been forcibly abducted and recruited into an armed group during the M23 war (2012–2014) were invited to a meeting. Following this meeting, interested participants were met on an individual basis, and the study procedure and confidentiality was explained in detail. All participants gave written informed consent.

The study protocol was approved by the Ethical Commission of the University of Konstanz (31/2016) and the governmental Social Fund of the DRC

Assessment measures were administered *via* structured clinical interviews conducted by seven local psychological interviewers who were specifically trained for the purpose of the study in a two-week training by two experienced clinical psychologists including the first author. The interviewers were fluent in the local dialect, Kiswahili. All measures were translated into Kiswahili and back translated into English to check for accuracy and discrepancies resolved through discussions between clinical psychologists and local translators. Further information regarding the training of interviewers and subsequent supervision is described in (59). The interviews took between 1.5 and 2.5 hours, in a confidential setting in Kibumba, Eastern DR Congo. Clients received transport money of 3,000 Congolese Francs (ca. 2 USD) and light refreshment for participation in the interview.

### Participants

**Figure 1:**
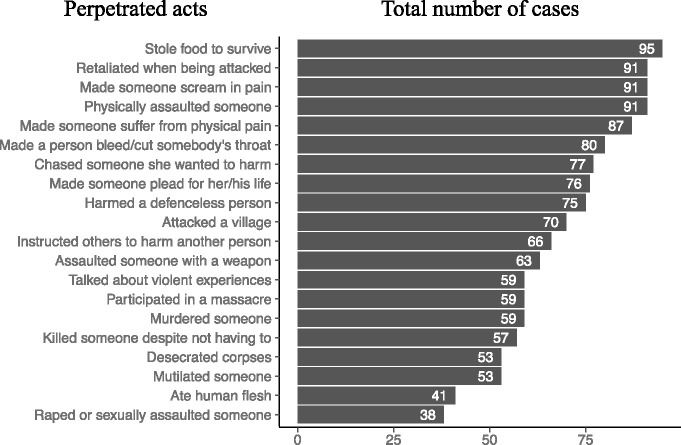
Major perpetrated acts ordered by the number of cases found among the study participants.

**Figure 2:**
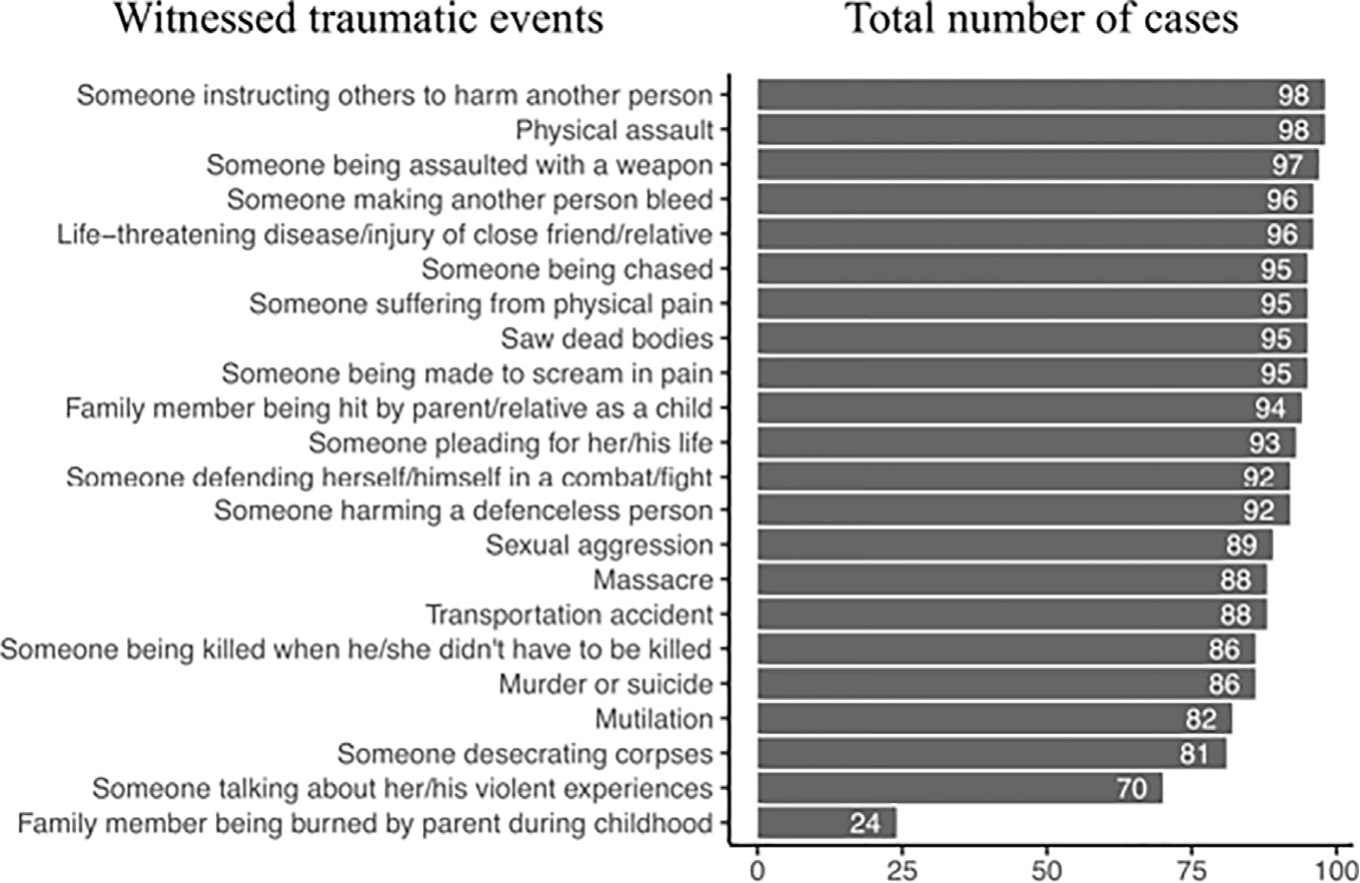
Major witnessed traumatic events ordered by the number of cases found among the study participants.

**Figure 3:**
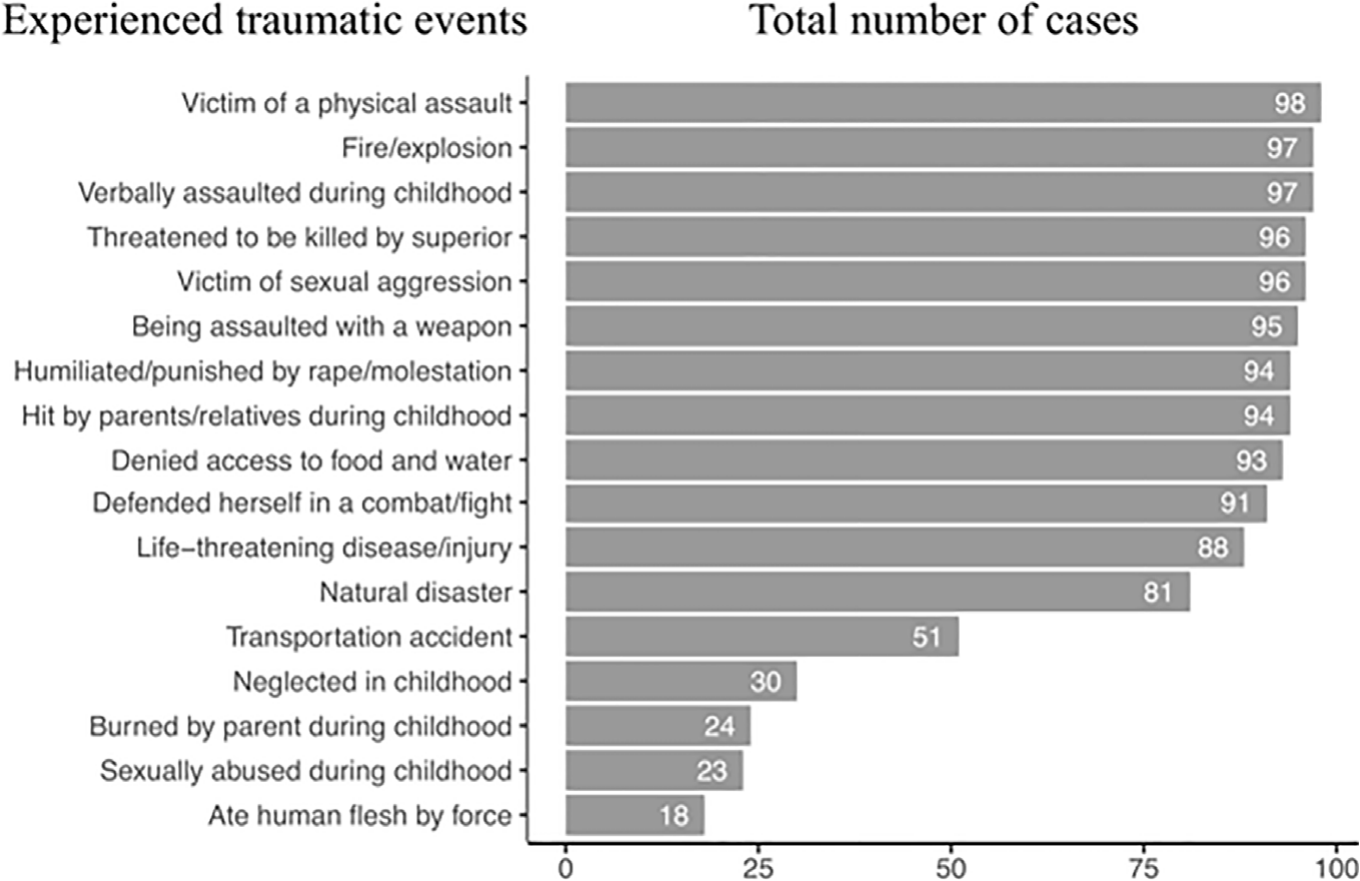
Major experienced events ordered by the number of cases found among the study participants.

In total, 99 young women who were either present at the community meeting or learned about the study from the NGO PAMI were interviewed; one was excluded since interviewers believed her to be younger than her stated age (treatment was provided outside of the study protocol). On average, they were 18 years old (range: 16–25), and the majority of them were single (77%). 19% were married or in a romantic relationship, 5% were divorced, and 2% widowed. Thirty-two (32%) reported to have at least one child. The women received on average 6 years of formal education (range: 0–12). Their ethnic origins were banyarbwisha (33%), banyarwanda (57%), munyarbwisha (3%), and other (7%). Time in captivity with armed group was between 1 and 2 years. The majority (78%) had been actively involved in combat, not only in in isolated incidents (12%) but also in two (19%), three (17%), or in several cases (29%). All but nine had been abducted before the age of 18 years. Lifetime prevalence of traumatic events in the form of perpetrated violent acts, witnessed and experienced events in this sample are shown in **Figures 1**–**3**, respectively. On average, participants encountered 32.3 traumatic events (range: 19–38).

### Measures

Demographic information included age, years of education, employment status, marital status, number of children, nature and age of recruitment into armed group, and frequency of direct combat.

#### Exposure to Trauma and Violence

This 44 item checklist of traumatic experiences was adapted from previous studies in similar populations in Eastern DRC [e.g., (20, 60, 61)]. It includes items relating to exposure to violence, including both direct experience and witnessing. The first seven items assess exposure to physical emotional sexual abuse and neglect during childhood, the remainder assess lifetime exposure to violence and other traumatic events. Twenty items of perpetrated violence are included. Sum scores were calculated for direct exposure and witnessing of traumatic events across the lifespan and for perpetrated acts of violence.

#### Outcomes

PTSD symptoms were assessed using the PTSD Symptom Scale Interview for DSM 5 [PSSI; (62)]. The PSSI assesses for PTSD according to the DSM-5 (63). Scores for each item range from 0 (not at all) to 4 (≤6 times a week/severe). Diagnosis was ascertained according to the manual, and sum scores were derived by adding all items of clusters B to E. Participants were instructed to answer in relation to an index trauma with a 1-month time frame. Previously, the DSM-IV and DSM-5 versions of the instrument have been used in Eastern DRC, with satisfactory psychometric properties (64). Interrater reliability and internal consistency were highly satisfying (ICC = .94, Cronbach’s α = .84) in this study.

Aggression was measured using the appetitive aggression scale [AAS; (65)]. The AAS is a 15 item semi structured interview which measures appetitive aggression according to the extent of agreement on a scale ranging from 0 (disagree) to 4 (agree) for items. A total sum score was used to measure severity of aggression. The instrument has been validated in similar contexts including those of the Eastern DRC and presented with high internal consistency (Cronbach’s α = .85) and interrater reliability (IRR = .98).

Current violent behaviour was measured using a 32 item checklist of aggressive or violent acts against partners, own children, and/or others within the preceding 3 months. This questionnaire has been used to assess armed combatants in Burundi (66). Items were scored according to whether or not they had occurred, and against whom, for each item. Sum scores provided a measure of current violent behaviour. Internal consistency and interrater reliably is highly satisfactory (Cohen’s κ = .97, Cohen’s α = .93). To calculate a unifying measure of current violent behaviour, accounting for the presence of children and partners, we used the following formula: (aggression_others_ + aggression_children_ + aggression_partner_)/(1 + children_presence_ + partner_presence_), where aggression stands for the sum scores of each subscale and children_presence_ and partner_presence_ stand for the presence (1) or not (0) of children and partners, respectively.

### Statistical Analyses

To test for the effects of AAS, PTSD, and trauma on current violent behaviour, general linear models were implemented in R 3.5.1 (67). First, we tested for the effect of interactions among predictors. Statistical significance of interactions was assessed by ANOVA tests comparing a model with against a model without the interaction in question. Since we did not find significant interactions in our data, we built new models including only the combination of the three main effects AAS, PTSD, and trauma. After model fitting, we compared models based on Akaike’s information criterion (AIC) and adjusted-R^2^. This allowed us to specifically observe whether psychopathological qualities (AAS and PTSD) and lifetime experience (trauma) independently affected current violent behaviour on our study sample. Significance of predictors was also assessed with ANOVA tests as described above. Cases with missing values above 10% (per instrument) were excluded in the final analysis, whereas those with 10% or less containing missings were imputed by predictive mean matching. To calculate current violent behaviour, the measures “children_presence_” and “partner_presence_” were also imputed using predictive mean matching. Imputation was implemented with the R package mice 3.3.0 (68).

## Results

Almost all of the women had been sexually assaulted or raped. They had also been physically assaulted, assaulted with a weapon, and threatened with being killed by their superior during the period with the armed group. High levels of perpetration of violence were evident (**Figure 1**), with almost all of the women acknowledging having made someone scream in pain and over half having mutilated someone and desecrated corpses. Almost half of the women had also participated in the sexual assault or rape of others and had eaten human flesh. In some cases, women described having authority over others, with over 60% describing having instructed others to harm someone. Witnessed (**Figure 2**) and directly experienced events (**Figure 3**) further reveal the high amount of lifetime traumatic events experienced by the study participants. On average, they witnessed 17.7 (SD = 2.1, range: 9–20) and experienced 15.2 (SD = 1.9, range: 9–19) types of traumatic events and perpetrated 14.1 (SD = 5.0, range: 3–20) types of aggressive acts.

Additionally, clinical symptomatology was high. Participants had an overall mean PSSI sum score of 37 (SD = 10.8), mean AAS sum score of 23.6 (SD = 11.8), and mean current violent behaviour of 18.9 (SD = 6.9).

**Figure 4:**
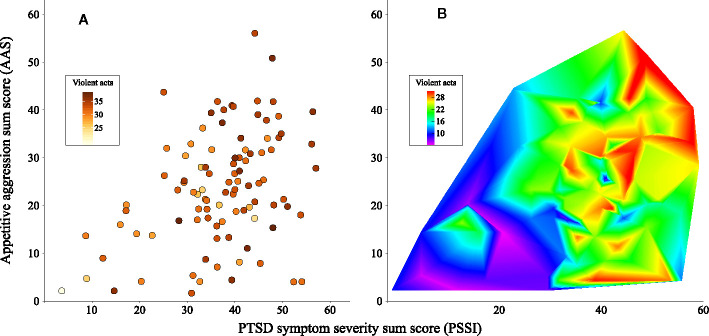
Effect of PTSD symptom severity (PSSI) and appetitive aggression (AAS) on current violent behaviour (violent acts). **(A)** Scatterplot showing individual observations. **(B)** Heatmap showing the intensity of violent acts as a function of PTSD and AAS. Violent acts are labelled with colours in both panels.

After model comparisons, the model with the best fit (adjusted-*R^2^* = 32.8%) included the AAS (F = 7.88, p = .006) and PSSI (F = 24.31, p <.001), showing that these two factors independently and positively affect current violent behaviour. Lifetime traumatic events (trauma) did not improve fit measures. **Figure 4** shows the relationships between AAS, PSSI, and current violent behaviour. **Table 1** presents model fits and summaries of significance tests of the variables in all models, and **Table 2** provides a summary of the best model.

**Table 1:** List of general linear models tested to assess the effect of AAS, PTSD and trauma on current violent behaviour by means of analysis of variance tests (ANOVAs).

Model	Model fit		ANOVA test to assess model effect significance					
Formula	AIC	Adj.-R^2^	*AAS*		*PSSI*		*Trauma*	
			F	p-value	F	p-value	F	p-value
*curr.viol.behav. ~ AAS + PTSD + trauma*	623.51	0.33	6.89	0.010	18.38	<0.001	0.77	0.381
**curr.viol.behav. ~ AAS + PTSD**	**622.32**	**0.33**	**7.88**	0.006	**24.31**	**<0.001**	not tested	not tested
*curr.viol.behav. ~ AAS + trauma*	639.02	0.21	13.81	<0.001	not tested	not tested	5.62	0.020
*curr.viol.behav. ~ PTSD + trauma*	628.44	0.29	not tested	not tested	26.21	<0.001	1.65	0.203
*curr.viol.behav. ~ AAS*	642.65	0.17	20.44	<0.001	not tested	not tested	not tested	not tested
*curr.viol.behav. ~ PTSD*	628.13	0.28	not tested	not tested	39.04	1.14E-08	not tested	not tested
*curr.viol.behav. ~ trauma*	650.32	0.10	not tested	not tested	not tested	not tested	11.67	0.001

bold, best model fit; grey background, variable not present in model formula; grey font, non-significant variable after ANOVA.

Model formula, model fit, and ANOVAs are shown for each model. Best model fit was based on AIC and the adjusted-R^2^. Trauma is the number of lifetime traumatic events.

**Table 2:** Final model was chosen based on best model fit (see **Table 1** ).

Model	Model fit				
Formula	AIC	Adj.-R^2^	F	p-value	
*curr.viol.behav. ~ AAS + PTSD*	622.32	0.33	24.9	<0.001	***
Model coefficients summary					
**Coefficient**	**Estimate**	**std. error**	**t**	**p-value**	
(Intercept)	5.03	2.09	2.4	0.017	*
*AAS*	0.15	0.05	2.8	0.006	**
*PTSD*	0.28	0.06	4.9	<0.001	***

Significance codes for p-value: ≤0.001***, ≤0.01, ≤0.05.

Model coefficients summary, including estimate, standard error, and t- and p-values are shown. Note that AAS and PTSD were significant effects explaining current violent behaviour.

## Discussion

This study documents the life experiences and mental health in a female sample of former child soldiers affected predominantly during the M23 insurgency in Eastern DRC and provides insight into the mechanisms that transfer war-related trauma into post conflict family and community violence. In line with earlier investigations of the United Nations Security Council (October 29, 2014), the results showed extremely high levels of traumatisation and perpetration of violence. Furthermore, the sample presented with high levels of appetitive aggression and PTSD symptom severity, which independently predicted the continuation of violence post conflict. Evidence-based psychotherapies for the treatments of PTSD and, recently, also appetitive aggression are available and effective, even in such challenging contexts like Eastern DRC [e.g. (59)]. The study highlights the importance of addressing the treatment needs of severely affected female survivors and provides important evidence for peacebuilding programmes in post conflict settings.

### Female (Child) Soldiers and Mental Health

Our findings demonstrate an extreme level of trauma exposure both in terms of frequency and atrocity and also a history of perpetration of severe violence, which was often forced but also in many cases adopted under the pressure of survival. While many of the women described their role in the group as “wives” of soldiers and had been abducted into sexual slavery, almost three quarters had also been involved in active combat during their period with the armed group. These young women, all abducted within a relatively short time frame by the same armed group for the M23 war, have experienced extreme exposure to violence and have been perpetrators of severe cruelty, often by force. They presented with the mental sequalae of these events– almost all participants fulfilled the diagnostic criteria for PTSD. Our study suggests former female (child) soldiers are a subgroup of survivors who require specific attention to address their clinical needs and to counter the effects on their social environment and society at large in the longer term.

### Current Aggressive Behaviour

In **Figure 4**, the relationship between PTSD symptoms, appetitive aggression and current violent behaviour is presented. Additionally, the models indicate that PTSD symptom severity and appetitive aggression independently predict current violent behaviour while the relevance of trauma exposure resides as these variables are added to the model. Previous findings have indicated the number of traumatic events underlying PTSD and appetitive aggression not only in (predominantly male) samples in DRC (56, 61) but also elsewhere [e.g., (1, 69, 70)]. Therefore, it is not surprising that the psychopathological variables outperform the underlying trauma exposure in the models. However, this is important since there are psychotherapies that have been proven effective for these problems. The other important finding from the regression model is that PTSD symptom severity and appetitive aggression independently predict ongoing violence – supporting firstly that two distinct “cycles of violence” may drive ongoing violent behaviour post conflict and secondly that both types of consequences – PTSD and appetitive aggression – need to be addressed in psychotherapy.

The “bi”-cycle of violence has previously been postulated for male samples (36) but might also be applicable to female survivors of trauma and violence. Elbert and colleagues (36) describe on one hand the sequelae of trauma and the experience of violence with PTSD, depression, and dissociation leading to more impulsivity and reactive aggressive behaviour and on the other hand perpetration (forced and/or voluntary) increasing appetitive aggression and psychopathy and leading to pro-active aggression. These findings from our theoretical background and the quantitative date converge with dominating narratives on site. For example, women who were reacting violently within the community post conflict reported being called “kisigira” (meaning “worthless” and associated with having been raped) by members of the community within the current setting, which may have directly triggered PTSD symptoms relating to experience of rape during captivity and caused extraordinarily strong aggressive responses not only partly impulsive but also goal directed and carefully planned (exaggerated by their appetitive aggressive traits).

With psychopathology explaining about 30% of variance in current violent behaviour, the importance of strengthening evidence from clinical psychology and psychotraumatology in order to adapt existing evidence-based treatments to this group and setting becomes evident. To this end, we have adapted Narrative Exposure Therapy in multiple trials with male former combatants (71–74) and have now applied it to a female sample with very promising results (59). Integrating a therapeutic component into peacebuilding interventions may decrease violence in high risk groups who would otherwise interfere with stabilisation initiatives through violent actions. Further research in this area is required.

Limitations of this study include the reliance of self-report of perpetration histories as well as current violence. These may be affected by social desirability. However, given the very high confirmations of perpetration of violence and high acknowledgement of current violence, social desirability does not appear to have unduly influenced reporting of aggression. More compellingly, the relationship between trauma exposure and clinical symptoms validates the outcome as does the regression model predicting current aggressive behaviour. Another limitation is that the generalisability of these findings to other combatants in other contexts is not known.

In conclusion, female former child soldiers experience high levels of trauma during their forced involvement in the armed group, both in form of victimisation and perpetration of violence. Psychological sequalae have a tremendous impact on their individual and social lives and enhance hostile dynamics and insecurity within the family and the wider community post conflict. More research is needed to establish evidence-based care and counter the rapidly accelerating cycle of violence in post conflict communities.

## Data Availability Statement

The datasets generated for this study are available on request to the corresponding author.

## Ethics Statement

The study was reviewed and approved by the ethics commission of the University of Konstanz. Written informed consent from the participants’ legal guardian/next of kin was not required to participate in this study in accordance with the national legislation and the institutional requirements.

## Author Contributions

KR, AK, SS and TE contributed to the study design and development. KR, SS and AC trained the interviewers. KR, AC and SS collected the data. KR and SS carried out preliminary statistical analysis. SC and AK carried out secondary analyses. KR, SC, and AK had full access to data and take responsibility for accuracy. KR drafted the manuscript. All authors contributed to the article and approved the submitted version. AK and TE provided supervision on all aspects.

## Conflict of Interest

The authors declare that the research was conducted in the absence of any commercial or financial relationships that could be construed as a potential conflict of interest.

The reviewer AM declared a shared affiliation, with no collaboration, with several of the authors, KR, AC, TE, and AK, to the handling editor at the time of review.
